# Axial Disease in Psoriatic Arthritis: how can we Define it, and does it have an Impact on Treatment?

**DOI:** 10.31138/mjr.33.1.142

**Published:** 2022-04-15

**Authors:** Clementina Lopez-Medina, Nelly Ziade

**Affiliations:** 1 Rheumatology Department, Reina Sofia University Hospital, Maimoindes Institute of Biomedical Research from Cordoba, Cordoba, Spain,; 2 Rheumatology Department, Cochin Hospital, Paris, France,; 3 Rheumatology Department, Saint-Joseph University, Beirut, Lebanon,; 4 Rheumatology Department, Hotel-Dieu de France Hospital, Beirut, Lebanon

**Keywords:** axial spondyloarthritis, axial psoriatic arthritis, differential diagnosis, treatment, imaging, outcomes, patient profile, personalised medicine

## Abstract

The Spondyloarthritis (SpA) represents a group of rheumatic inflammatory entities that share clinical, laboratory and imaging features, including Psoriatic Arthritis (PsA). Axial involvement may occur in up to 50% of patients with PsA (axPsA), causing inflammatory back pain, stiffness and changes on imaging. Whether axial SpA (axSpA) with psoriasis represents a distinct entity than axPsA is a matter of debate, since similarities and differences have been reported in terms of clinical expression and imaging. Patients with radiographically axPsA show lower prevalence of inflammatory b ack pain, lumbar and buttock pain in comparison with axSpA. In addition, imaging features differ between axPsA and axSpA, with less sacroiliitis in axPsA and more asymmetrical, chunky syndesmophytes which are predominant at the cervical spine location. Data on treatment efficacy and management recommendations are extrapolated from studies on axSpA, and only one published randomized clinical trial is dedicated specifically to axPsA to date.

## INTRODUCTION

The Spondyloarthritis (SpA) represents a group of rheumatic inflammatory entities that share clinical, laboratory and imaging features. The spectrum of SpA includes patients with a predominant axial involvement (axial SpA [axSpA]) and patients with a predominant peripheral involvement (peripheral SpA [pSpA]).^1^ Apart from articular symptoms, many SpA patients exhibit an array of extra-musculoskeletal manifestations, including psoriasis, anterior uveitis and inflammatory bowel disease (IBD). The presentation of these features leads to the classic classification into several subtypes, such as radiographic axial SpA (r-axSpA, previously known as ankylosing spondylitis (AS)), psoriatic arthritis (PsA), IBD-associated SpA and reactive arthritis (ReA).^2^

PsA is considered part of the spectrum of SpA, and is characterized by the involvement of the skin, peripheral joints and axial skeleton. Although peripheral arthritis is the most frequent clinical feature in PsA patients,^3^ axial involvement may occur in up to 50% of patients with PsA (ie, axPsA), causing inflammatory back pain (IBP), stiffness and changes on imaging.^4^ However, whether axS-pA with psoriasis represents a distinct entity than axPsA is a matter of debate, since similarities and differences have been reported in terms of clinical expression and imaging.

In this review, we aim to summarize the definition, clinical presentation, imaging and treatment response of axPsA and also to highlight the similarities and differences with axSpA.

## DEFINITION

Up till now, there is not a clear definition of axPsA. Indeed, different terms have been used to refer to axial involvement in the context of PsA,^5^ such as “axial psoriatic arthritis”,^6^ “psoriatic spondyloarthropathy”,^7^ and “psoriatic spondylitis”.^8^ This lack of a definition has challenged the alignment of clinical studies in this area, raising the necessity of a consensus on the definition of axial involvement in these patients. Previous criteria proposed for defining axPsA vary from minimal radiographic evidence (such as isolated unilateral grade 2 sacroiliitis)^7,9^ to the modified New York criteria used for the diagnosis of AS,^10^ which include both clinical criteria and stringent radiographic criteria of at least bilateral grade 2 sacroiliitis or unilateral grade 3 or 4 sacroiliitis.^11^ As a consequence of this broad spectrum of proposed criteria, the prevalence of axPsA ranges from 25% to 75%.^12^ Moreover, the prevalence of axial disease in patients with PsA varies depending on the disease duration, occurring in 5–28% of patients with early disease, and in 25–70% of patients with longstanding PsA,^13^ suggesting that axial disease typically develops at a later stage in the disease course.

## CLINICAL CHARACTERISTICS

The major clinical manifestation of spinal inflammation is IBP, characterized by insidious onset, improvement with exercise and nocturnal pain.^14^ However, IBP may appear in patients with axSpA and patients with r-axSpA. While IBP is essential for a diagnosis of r-axSpA,^15^ only 45% of patients with radiographically axPsA have this symptom.^4^ In addition, AS group shows more symptoms of thoracic and buttock pain than the axPsA group,^16^ suggesting that IBP might not be a good criterion for identifying axPsA patients.^17^

Among patients with PsA, men and females show similar prevalence of axial involvement.^16,17^ However, patients with axPsA are typically younger than those with PsA without axial disease^16–18^ and have a younger age at disease onset.^16,18,19^

The HLA-B27 antigen has a strong association with sacroiliitis in both patients with axSpA and PsA. Despite a variable prevalence of HLA-B27 reported in cases with axPsA, data are consistent showing that this antigen is more prevalent in axPsA (19% to 49%) rather than in pure PsA (3.8% to 19%).^16,17,18,19^ Data regarding extra-articular manifestations in axPsA are scarce. Few studies reported that patients with axial involvement were more likely to have a history of IBD^16^ and uveitis.^19,20^ Moreover, uveitis was more prevalent in axPsA with HLA-B27 positive than negative.^21^

## PATIENT-REPORTED OUTCOMES

Up till now, no specific Patient-Reported Outcomes (PROs) of axial disease activity and function have been developed for PsA. Measures designed for use in axSpA have been applied on the evaluation of axPsA patients, such as the Bath Ankylosing Disease Activity Index (BASDAI),^22^ the Ankylosing Spondylitis Disease Activity Score (ASDAS)^23^ and the Bath Ankylosing Spondylitis Functional Index (BASFI).^24^ However, the BASDAI was found to not correlate well with external indicators of axial disease activity, so the validity of the BASDAI as a tool for the measurement of axial disease activity in axPsA is questionable.^13,25,26^ The comparison of disease activity between axPsA and axSpA in terms of PROs showed variable data: some studies demonstrated a higher disease activity measured by the BASDAI^18,19^ and ASDAS18 in axPsA patients in comparison with axSpA, while other studies reported a similar disease activity.^27^

## IMAGING IN AXIAL PSORIATIC ARTHRITIS

### Prevalence of radiologic abnormalities

The prevalence of radiologic abnormalities in axPsA is variable between studies, depending on the examined population and the time of assessment during the disease course.

Interestingly, radiologic abnormalities can be detected on routine radiographs in patients without axial symptoms. In a prospective study from the University of Toronto Psoriatic Arthritis longitudinal database, among axPsA patients who had radiographic evidence of spinal disease at their initial assessment, and in whom there was a minimum follow-up of 30 months, 35% had no axial symptoms.^27^ The follow-up at 10 years from the same database showed that more patients (44%) had no axial symptoms.^28^ Similarly, a more recent prospective single-centre cross-sectional observational study (Axial Disease in Psoriatic Arthritis study) showed that 25% of the patients with PsA and radiographical axial disease (and a mean duration of clinical disease 18 years) had a symptomatically silent axial disease.^16^

In these axial-asymptomatic patients, these radiologic abnormalities’ significance remains undetermined.^19^

Regarding radiographic progression, longitudinal data is scarce. The Toronto database^29^ found that 51.7% of the patients with no evidence of sacroiliitis at baseline developed grade 2 or 3 sacroiliitis at 10 years; and 52% with grade 2 progressed to grade 3 or 4 sacroiliitis. Regarding the spine, approximately 15% to 20% of patients without syndesmophytes at study entry had developed syndesmophytes after 10 years. HLA-B27-positive ax-PsA patients developed more lumbar syndesmophytes in comparison with HLA-B27-negative axPsA patients.

### Imaging of the sacroiliac joints

#### X-rays of the sacroiliac joints

Sacroiliitis is the hallmark feature of axSpA and a main entry point to the ASAS classification criteria.^30^ However, it is not a frequent feature of axPsA, as up to 35% will present with radiologic spondylitis without sacroiliitis.^5,16^ The absence of radiographic sacroiliitis was associated with less HLA-B27 positivity.^16^

Moreover, even when present, SIJ involvement tends to be asymmetrical and more unilateral as compared with axSpA and to develop less frequently into complete ankylosis (43% *versus* 15%, adjusted OR 2.96; 95% CI 1.42 to 6.15).^9,16,19^

#### MRI of the sacro-iliac joints

A study of 103 patients with PsA found that only 38% had MRI sacroiliitis.^31^ However, they found no correlation with the clinical signs or the HLA-B27 status, using a binary definition of sacroiliitis. In contrast, another study with 76 patients with symptomatic axPsA found a significant correlation between the extent of bone marrow edema, using a semi-quantitative score, and the HLA-B27 status.^32^ Compared to axSpA, total MRI scores (sacroiliac joints and lumbar spine) were lower in axPsA and were negatively correlated with the HLA-B27 status.^32^

Therefore, including sacroiliitis in an eventual axSpA classification criteria system would be very challenging.

### Imaging of the spine

#### X-rays of the spine

First, the location of spine involvement is different between axSpA and axPsA. AxSpA presents changes predominantly in the lumbar spine, whereas axPsA has a predilection for involving the cervical spine, which may involve the posterior elements as well.^33,34^ This cervical involvement may be the only axial symptom and is mirrored by a deterioration in cervical mobility over a 10-years follow-up in Toronto longitudinal study.^4^

**Figure 1:**
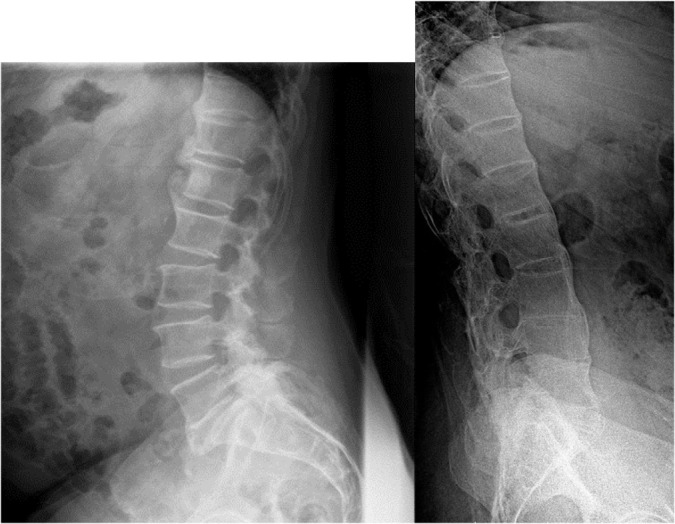
Coarse and “chunky” syndesmophytes in a male patient with Psoriatic Arthritis and axial involvement (A), and typical “bamboo spine” in a male patient with axial Spondyloarthritis (B).

Second, the morphology of syndesmophytes is also distinct between both diseases. In axSpA, syndesmophytes are marginal, symmetrical, and well-delimited, whereas they are asymmetrical, coarse, and “chunky” in axPsA (**Figure 1**). This syndesmophyte morphology raises a significant clinical challenge of differential diagnosis with a relatively common condition, diffuse idiopathic skeletal hyperostosis (DISH). In fact, both diseases can even coexist with an 8.3% prevalence of DISH in patients with PsA similar to the general population, which may raise difficulties in making therapeutic choices.^35^ Another differential diagnosis to consider is “simple” osteoarthritis, as osteophytes can be frequent in axPsA patients in relation to their older age compared to axSpA.

Third, compared to axSpA, bridging syndesmophytes develop less frequently in axPsA (23% *versus* 10%, adjusted OR 2.78; 95% CI 1.49 to 5.18).^16^ Involvement of the apophyseal joints is also less frequent in patients with axPsA, which may explain why these patients have less restricted axial mobility compared to patients with axSpA.^6^ (**Table 1**).

**Table 1:** Radiologic features in axPsA Aversus axSpA.

	**Axial SpondyloArthritis**	**Axial Psoriatic Arthritis**

Sacroiliitis	Hallmark of disease	Less frequent feature
	More severe	Less severe, less complete ankylosis
	Symmetrical	Asymmetrical
		More frequent in HLA-B27+ patients

Spondylitis	Usually occurs after sacroiliitis	Can occur without sacroiliitis in 35%
	More frequent in the lumbar spine	More frequent in the cervical spine
	Syndesmophytes are marginal, symmetrical and well-delimited	Syndesmophytes are asymmetrical, «chunky,» and coarse
	More bridging syndesmophytes	Less bridging syndesmophytes
	More involvement of the apophyseal joints	Less involvement of the apophyseal joints
	Higher global severity score (PASRI)	Lower global severity score (PASRI)

PASRI: Psoriatic Arthritis Spondylitis Radiology Index Score.

**Table 2:** Evidence on biologic therapies in patients with psoriatic arthritis and axial manifestations.

	**Secukinumab**	**Guselkumab**	**TNF inhibitors**
Study Design	RCT	Post-hoc analysis of 2 RCTs	Single-centre longitudinal observational study
Definition of axial involvement	Spinal pain VAS ≥40/100 and BASDAI score ≥4	Imaging-confirmed sacroiliitis	IBP (ASAS criteria) and/or radiological axial involvement
Number of patients	498	312	58
HLA-B27 positive	33%	30%	22.4%
Primary Endpoint	ASAS20	BASDAI and ASDAS	BASDAI50
Time of assessment	12 weeks	24 weeks	12 months
Other endpoints	ASAS40, BASDAI50, Spinal VAS, HAQ-DI, FACIT-Fatigue, ASAS-HI and ASDAS-CRP		CPDAI, DAPSA, PR, MDA
Outcome	63–66% ASAS20 improvement with secukinumab versus 31% with placebo	Clinically important improvement in ASDAS (change ≥1.1) observed in 55% on Guselkumab versus 28% in placebo arm	BASDAI50 achieved in 31.2% of patients
Reference	Baraliakos 2020	Mease 2020	Lubrano 2016

ASAS: Assessment of SpondyloArthritis international Society, ASAS-HI: ASAS Health Index, ASDAS: AS disease activity score, BASDAI: Bath Ankylosing Spondylitis Disease Activity Index, BASFI: Bath Ankylosing Spondylitis Functional Index, BASMI: Bath Ankylosing Spondylitis Metrology Index, CASPAR: classification criteria for psoriatic arthritis, CPDAI: Composite Psoriatic Disease Activity Index, CRP: C-Reactive Protein, DAPSA: Disease Activity Index for Psoriatic Arthritis, ESR: Erythrocyte Sedimentation Rate, FACIT: Functional Assessment of Chronic Illness Therapy, HAQ: Health Assessment Questionnaire, HAQ-DI: HAQ Disability Index Score, IBP: Inflammatory Back Pain, PR: partial remission, MDA: Minimal Disease Activity, MRI: Magnetic Resonance Imaging, PGA: Patient Global Assessment, RCT: Randomized Controlled Trial, RLDQ: Revised Leeds Disability Questionnaire, SIJ: Sacro-Iliac Joints, VAS: Visual Analogue Score.

The radiographic scores which are used in axSpA, such as the Bath Ankylosing Spondylitis Radiology Index (BASRI) and the Modified Stokes Ankylosing Spondylitis Spinal Score (mSASSS), have a good correlation with metrology and clinical measures in axPsA as well [36]. Moreover, a score explicitly designed for axSpA and taking into account the cervical zygapophyseal involvement, the Psoriatic Arthritis Spondylitis Radiology Index (PASRI), correlated well with both metrology and patient-reported outcomes.^37^ Globally, radiographic axial disease was more severe in axSpA than axPsA, according to the PASRI score (adjusted incidence risk ratio 1.13; 95% CI 1.09 to 1.19).^6,10,16^

#### MRI of the spine

In the Toronto cohort, spinal MRIs, requested mainly for axial inflammatory symptoms, found abnormalities compatible with SpA in 44.6%, including bone marrow edema in 18.5% and erosions in 15.6%.^38^

In general, radiologic abnormalities in axPsA are more subtle than the features in axSpA; therefore, their value in eventual classification criteria is also debatable.

## THERAPEUTIC EVIDENCE IN AXIAL PSORIATIC ARTHRITIS

### International recommendations

Apart from the recommendations for treating axSpA in general,^39,40^ there are currently no recommendations dedicated for the treatment of axial disease, specifically in patients with PsA.

The EULAR 2019 recommendations for the management of PsA reserved a statement for patients with predominantly axial disease, which is active and has an insufficient response to non-steroidal anti-inflammatory drugs (NSAIDs). In that case, “therapy with a biological disease-modifying drug (bDMARD) should be considered, which according to current practice is a Tumor Necrosis Factor inhibitor (TNFi); when there is relevant skin involvement, Interleukin-17 (IL-17) inhibitor may be preferred”.^41^

Also, the GRAPPA 2015 recommendations for PsA, which tackled the treatments according to disease domains, derived the recommendation for axial disease from the data on axSpA. They stated that “for patients with axial symptoms that have not responded to NSAIDs, physiotherapy, and sacroiliac joint injections (when appropriate), initiation of TNFi is recommended; DMARDs are not effective for the treatment of diseases in this domain.” In case of inadequate response to TNFi, they added a conditional recommendation for using secukinumab or ustekinumab (based on a phase 3 RCT and an open-label proof-of-concept trial published at that time, respectively).^42^

### Corticosteroids: a differential response between axPsA and axSpA

In a prospective, open-labeled, controlled pilot study, corticosteroids’ performance (single intramuscular dose of Triamcinolone acetonide 80 mg) in patients with axPsA was compared to those with active ankylosing spondylitis (AS) and a control group of patients with non-inflammatory lower back pain. Patients had bone marrow edema on MRI of the sacroiliac joints and a clinically active disease defined as inflammatory back pain (fulfilling ASAS criteria), with spinal pain score (numerical rating scale 0–10) ≥4 and BASDAI score ≥4 despite taking NSAIDs. In total, 40 patients were recruited (15 with axPsA, 15 with AS, and 10 controls). At week 2, axial inflammation in patients with PsA responded significantly better to corticosteroids than in patients with AS (mean ASDAS improvement in patients with axPsA compared to patients with AS (1.43 ± 0.39 vs. 1.03 ± 0.30, p = 0.004), and also when compared to controls (p < 0.001), supporting the evidence that axSpA and AS are two different entities and may have differential responses to one or another treatment.^43^

### Secukinumab: the MAXIMISE trial

To date, there is only one published randomized controlled trial (RCT) specifically studying the efficacy of biologics in the treatment of PsA patients with axial manifestations, the Managing AXIal Manifestations in psorIatic arthritis with Secukinumab (MAXIMISE) trial conducted by Baraliakos et al.^44^ (**Table 1**). The study included 498 patients diagnosed with PsA and classified by the CASPAR criteria, with spinal pain Visual Analogue Score ≥40/100 and Bath Ankylosing Spondylitis Disease Activity Index (BASDAI) score ≥4 despite the use of at least two NSAIDs. The patients were not required to fulfill the axSpA modified New York or ASAS classification criteria to avoid restriction to axSpA patients with psoriasis. The objective of the study was to evaluate the efficacy of Secukinumab, a fully human monoclonal antibody that directly inhibits IL-17A and which is already used for the treatment of axSpA, PsA, and psoriasis in the management of the axial manifestations of PsA,^45–48^ using the ASAS20 at week 12 as a primary outcome. Patients were randomly assigned to Secukinumab 300 mg, Secukinumab 150 mg, or placebo arms weekly for 4 weeks and every 4 weeks thereafter. The patients had an established diagnosis of PsA with symptoms for around 7 years on average, their mean age was 46.6 years, and 49.4% were men. Around 60% of patients had a positive MRI with inflammation in the spine and/or SIJ. HLA-B27 status, available for 261 patients, was positive in 33% of the patients, 90.8% of the patients had current psoriasis at inclusion, and the mean baseline BASDAI score was 7.3. The results showed a significant improvement in the ASAS20 response with Secukinumab 300 mg and 150 mg compared to placebo (63% and 66% *versus* 31% placebo). The OR (95% CI) for reaching ASAS20 response in the comparison of Secukinumab 300 and 150 mg versus placebo, using a logistic regression model, was 3.8 (2.4 to 6.1) and 4.4 (2.7 to 7.0; p<0.0001). Secukinumab also demonstrated significant improvements across the secondary clinical (ASAS40, BASDAI50, Spinal VAS, HAQDI, FACIT-Fatigue, ASAS-HI, ASDAS-CRP) and imaging (Berlin MRI score) endpoints. At week 12, placebo patients were re-randomized to Secukinumab 300/150 mg, with 85% of them completing the trial through week 52. The positive results observed at week 12 were sustained through week 52. Moreover, patients on placebo who switched to Secukinumab 150 mg or 300 mg at week 12 improved rapidly and considerably across all assessed efficacy endpoints. The study showed similar clinical responses in the MRI positive patients (for approximately 60% of the trial population) and the overall population regardless of MRI status at baseline.

An indirect informal comparison with the results of Secukinumab in axSpA shows that the response in axP-sA is within a similar range. The study by Baeten et al.^45^ on ankylosing spondylitis, showed an ASAS20 response of 61% in the Secukinumab 150 mg arm (versus 28% in the placebo arm), and the study by Deodhar et al.^47^ on non-radiographic axSpA showed an ASAS20 response of 56.8% in the Secukinmab 150 mg arm (versus a high 45.7% in the placebo arm), both at week 16.

### Guselkumab

A posthoc analysis of two phase 3 studies, DISCOVER1 and DISCOVER2 [49,50] explored the efficacy of Guselkumab, a human monoclonal antibody that specifically binds to the p19-subunit of IL-23 already approved in psoriasis and PsA, for the improvement of axial symptoms in PsA patients.^51^ Patients with active PsA and imaging-confirmed sacroiliitis were randomized to Gulselkumab or placebo up to week 24 then switched to Guselkumab and followed up until week 60. Sacroiliitis was confirmed either by documented prior imaging or pelvic radiograph at screening and was available in 312 of 1120 patients with PsA. HLA-B27, available in 61% of the patients, was positive in 30%. BASDAI- and ASDAS-related axial efficacy endpoints were assessed at week 24 and showed a significant improvement for both measures (BASDAI mean change from baseline −2.7 in the Guselkumab arms and −1.3 in the placebo arm, p<0.001; ASDAS mean change from baseline −1.4 in the Guselkumab arms and −0.7 in the placebo arm, p<0.001). At week 24, a clinically important improvement in ASDAS (change ≥1.1) was observed in 55% of the patients taking Guselkumab every 4 weeks, versus 28% in the placebo arm (p<0.001) and a major improvement in ASDAS (change ≥2.0) was observed in 29% of the patients taking Guselkumab every 4 weeks, versus 9% in the placebo arm (p<0.001). The improvement of the axial symptoms was maintained through week 52 and was observed irrespective of HLA-B27 status.

### TNF inhibitors

A longitudinal observational monocenter study, based on a clinical practice setting, enrolled patients fulfilling the CASPAR criteria and treated with TNFi agents Adalimumab, Etanercept, and Golimumab and prospectively followed them every 4 months for 1 year. The definition of axPsA was classified according to the criteria proposed by ASAS for inflammatory back pain and/or radiological axial involvement.^14^ BASDAI50 (defined as BASDAI 50% relative or absolute change of 20 mm and expert opinion in favor of continuation) was achieved in 31.2% of the patients. No difference was found among the 3 TNFi.^52^ This response seems to be lower than the one usually observed with the TNFi in the treatment of axSpA, as shown by a review indicating a BASDAI50 response in the 50–60% range,^53^ although no direct comparison can be made based on the available data.

Another multicenter observational study evaluated Etanercept in 32 patients with PsA (classified by the CASPAR criteria) and axial manifestations. Effectiveness of Etanercept was observed in 72% of patients for the BASDAI50 (defined as BASDAI 50% relative or absolute change of 20 mm and expert opinion in favor of continuation, p<0.001) and in 68% for the BASFI (p<0.001).^54^ However, this result should be interpreted very carefully due to the low number of patients and the observational nature of the study.

In conclusion, axial involvement in PsA manifests clinically and radiographically differently than in axSpA, although there is still not a consensus definition of axial involvement in these patients. Data on treatment efficacy and management recommendations are extrapolated from studies on axSpA, since only one RCT is dedicated specifically to axPsA to date. Future studies focused on axPsA are needed to define axial involvement in PsA and to elucidate the efficacy of different drugs in these patients.
